# Antimicrobial treatment practices among Ugandan children with suspicion of central nervous system infection

**DOI:** 10.1371/journal.pone.0205316

**Published:** 2018-10-09

**Authors:** Elizabeth Kemigisha, Deborah Nanjebe, Yap Boum, Céline Langendorf, Said Aberrane, Dan Nyehangane, Fabienne Nackers, Yolanda Mueller, Rémi Charrel, Richard A. Murphy, Anne-Laure Page, Juliet Mwanga-Amumpaire

**Affiliations:** 1 Epicentre Mbarara Research Centre, Mbarara, Uganda; 2 Mbarara University of Science and Technology, Mbarara, Uganda; 3 Epicentre, Paris, France; 4 Microbiology Laboratory, Créteil Hospital, France; 5 Centre de recherche et de développement Policlinique Médicale Universitaire, Lausanne, Switzerland; 6 EPV Emergence des Pathologies, UMR D 190, Marseille, France; 7 Division of Infectious Diseases, Los Angeles Biomedical Research Institute at Harbor–UCLA Medical Center, Torrance, CA, United States of America; Washington University School of Medicine in St. Louis, UNITED STATES

## Abstract

Acute central nervous system (CNS) infections in children in sub-Saharan Africa are often fatal. Potential contributors include late presentation, limited diagnostic capacity and inadequate treatment. A more nuanced understanding of treatment practices with a goal of optimizing such practices is critical to prevent avoidable case fatality. We describe empiric antimicrobial treatment, antibiotic resistance and treatment adequacy in a prospective cohort of 459 children aged two months to 12 years hospitalised for suspected acute CNS infections in Mbarara, Uganda, from 2009 to 2012. Among these 459 children, 155 had a laboratory-confirmed diagnosis of malaria (case-fatality rate [CFR] 14%), 58 had bacterial infections (CFR 24%) and 6 children had mixed malaria and bacterial infections (CFR 17%). Overall case fatality was 18.1% (n = 83). Of 219 children with laboratory-confirmed malaria and/or bacterial infections, 182 (83.1%) received an adequate antimalarial and/or antibiotic on the day of admission and 211 (96.3%) within 48 hours of admission. The proportion of those receiving adequate treatment was similar among survivors and non-survivors. All bacterial isolates were sensitive to ceftriaxone except one *Escherichia coli* isolate with extended-spectrum beta-lactamase (ESBL). The observed high mortality was not a result of inadequate initial antimicrobial treatment at the hospital. The epidemiology of CNS infection in this setting justifies empirical use of a third-generation cephalosporin, however antibiotic resistance should be monitored closely.

## Introduction

Acute infections of the central nervous system (CNS) in children are usually severe and associated with high case fatality rates (CFR; 10–35%). Up to 15% have major neurological sequelae [1–3]. Late and/or inadequate treatment of bacterial meningitis is associated with poor outcome [4,5]. To reduce such outcomes, CNS infections should be treated with an appropriate antimicrobial agent as early as possible [6]. Empiric antibiotic treatment is the standard of care for suspected bacterial CNS infections before the pathogen and its sensitivity to antimicrobials are confirmed by laboratory diagnosis. Inadequate treatment can result from antibiotic resistance or from inappropriate antibiotic choice for drug-sensitive bacterial infections.

In sub-Saharan Africa, factors associated with high CFR of CNS infections include suboptimal health care access and late presentation [7]. Furthermore, the clinical diagnosis of bacterial meningitis is particularly challenging given the high prevalence of other infections presenting with a similar clinical picture, such as cerebral malaria. In rural areas, health workers first consulted are often of a lower cadre and require critical supervision to differentiate between competing diagnoses [8,9]. Underutilization of lumbar puncture and poor access to microbiology services in many low-income countries contribute to poor knowledge of the local aetiology of bacterial meningitis and antibiotic resistance patterns [10].

Confronted with high mortality, especially within the first 48 hours of admission, clinicians in low income countries rely on empiric antimicrobial treatment for the management of children with suspected CNS infections, a strategy that is increasingly threatened by reduced antimicrobial susceptibility. Here we describe empiric antimicrobial treatment practices, antibiotic resistance and treatment adequacy in children with abnormal CNS symptoms and signs in the presence of bacterial or malaria infection in a regional referral hospital in Mbarara, Uganda. We focus on malaria and bacterial infections because of the limited ability to diagnose and assess treatment for viral infections. This is a secondary analysis of data collected within a study of the aetiology of suspected CNS infections published elsewhere [11].

## Materials and methods

### Study population

The study took place in two hospitals, Mbarara Regional Referral Hospital (MRRH) and Holy Innocents Children’s hospital (HICH) in Mbarara, located 300 km south-west of the capital Kampala. The prevalence of malaria in the Mbarara district ranges from 3% in urban areas to 23% in rural areas during the rainy season [12].Children between two months and 12 years admitted with suspicion of acute CNS infection based on fever or history of fever in the past 48 hours and recent onset of at least one sign of CNS involvement. CNS symptoms and signs included: non-traumatic reduced level of consciousness, prostration, hypertonia/hypotonia, irritability, severe headache, photophobia, neck stiffness, bulging fontanelle, prolonged seizures, focal neurological signs, purpura or Cheyne stokes breathing. Children who met the criteria above were enrolled into a prospective descriptive cohort study between August 2009 and October 2012. A more complete description of the study population and methods is provided elsewhere [11].

### Clinical and laboratory investigations

Socio-demographic and clinical characteristics of the participants including age, gender, HIV exposure, history and type of previous treatment as well as clinical information at admission and discharge and treatment received were recorded in a standardized case report form [11]. Diagnosis at admission was based on clinical examination and systematic testing with a rapid diagnostic test for malaria (SD-Bioline Malaria Antigen P.f/Pan, Standard Diagnostics, Korea).

Final diagnosis was informed by biological investigations of blood and cerebrospinal fluid (CSF). Sample collection was performed systematically upon inclusion. Venous blood samples were collected aseptically into two single bottle blood-culture system (Signal Blood Culture System, Oxoid) and into EDTA tubes for hematological analyses. CSF was aseptically obtained by lumbar puncture into sterile CSF tubes. To diagnose bacterial pathogens, blood and CSF were analyzed at the Epicentre research laboratory on site following standardized bacteriology procedures [11]. Culture, staining, biochemical testing were used to identify any bacterial pathogens present. Drug susceptibility testing (DST) was done following the Comité De L’antibiogramme De La Société Francaise De Microbiologie (CA-SASFM 2006) guidelines [13]. We used the Kirby-Bauer disc diffusion method and E-tests to determine drug clearance zones and minimum inhibitory concentrations (MICs), respectively.

In addition, real-time polymerase chain reaction (qPCR) was performed systematically on CSF to look for the following bacterial pathogens: *S*. *pneumoniae*, *H*. *influenzae* type b (Hib), *Salmonella*, *Listeria monocytogenes*, as well as viral pathogens not presented here. These qPCRs were carried out initially at Epicentre Research Laboratory, Mbarara and later at APHM Laboratory, Marseille, France, on CSF stored and transported at -80°C following standardized procedures [11].

Thick and thin blood smears were prepared from EDTA blood, Giemsa-stained and examined by microscopy as recommended by the World Health Organization (WHO) to confirm malaria infection [14].

### Description of treatment guidelines

Patients were treated by the attending ward clinician according to the treatment protocols of the pediatric wards of the MRRH and HICH, where the empiric treatment of meningitis is benzyl penicillin (Penicillin G) at 50,000 IU/kg every 6 hours combined with ceftriaxone at a dose of 100 mg/kg/day for 10–14 days modified according to results for culture or sensitivity. Treatment for severe malaria changed in the course of the study (September 2011) from intravenous quinine to intravenous artesunate, 3mg/kg at hour 0, 12 and 24 for a minimum of 48 hours followed by oral artesunate/lumefantrine (20/120mg) for a total of 3 days once the child can tolerate oral treatment [15]. There were no established hospital protocols to guide empiric treatment for bacteremia or concomitant antibiotic treatment for severe malaria.

### Definitions and data analysis

*Bacterial meningitis* was considered if a bacterial pathogen was detected in the CSF by culture, qPCR or Gram staining, whereas *bacteremia* was concluded if a bacterial pathogen was detected in blood but not in the CSF.

*Empiric treatment* was defined as the first treatment received after admission.

*Adequate treatment* was defined as treatment with antimalarial for a confirmed laboratory diagnosis of malaria and/or at least one appropriate antibiotic for bacterial infection according to the DST results (or extrapolated from overall DST results if DST results were not available for a specific isolate)–regardless of the duration of treatment and possible over-treatment.

*Insufficient treatment duration* was defined as an antimalarial treatment given for less than 3 days or antibiotic treatment given for less than 5 days for bacteremia or 10 days for bacterial meningitis, or for less than the number of days of hospitalization for those who died or were discharged against medical advice before the recommended treatment duration.

*Excessive treatment duration* was defined as an antimalarial given for more than 8 days or an antibiotic treatment given for more than 21 days for bacterial infections. The rationale for 21 days was to adequately treat for meningitic syndromes that require an extended treatment period such as infections resulting from *Enterobacteriaceae* or *Listeria monocytogenes*.

Double data entry and data validation was done using EpiData Entry software (version 3.1, EpiData Association, Odense, Denmark) and analysis was performed using Stata (version 12, College Station, Texas, USA).

Results were analyzed using descriptive statistics to obtain frequencies and proportions. Differences between groups were tested at 5% significance level. The analysis of antimicrobial treatment practices focused on children with a laboratory-confirmed malaria and/or bacterial infections with CNS involvement.

### Ethics

Ethical approval was obtained from Mbarara University Research and Ethics Committee (MUIRC 1/7), the Uganda National Council for Science and Technology (HS 584) and the Comité de Protection des Personnes, Ile de France XI, Saint-Germain en Laye France(reference 09013). Written informed consents for study participation were obtained from participants’ parents or legal guardians. All participants were provided with treatment free of charge. The study was in accordance with the ethical standards of the Helsinki Declaration.

## Results

### Description of the study population

**Fig 1:**
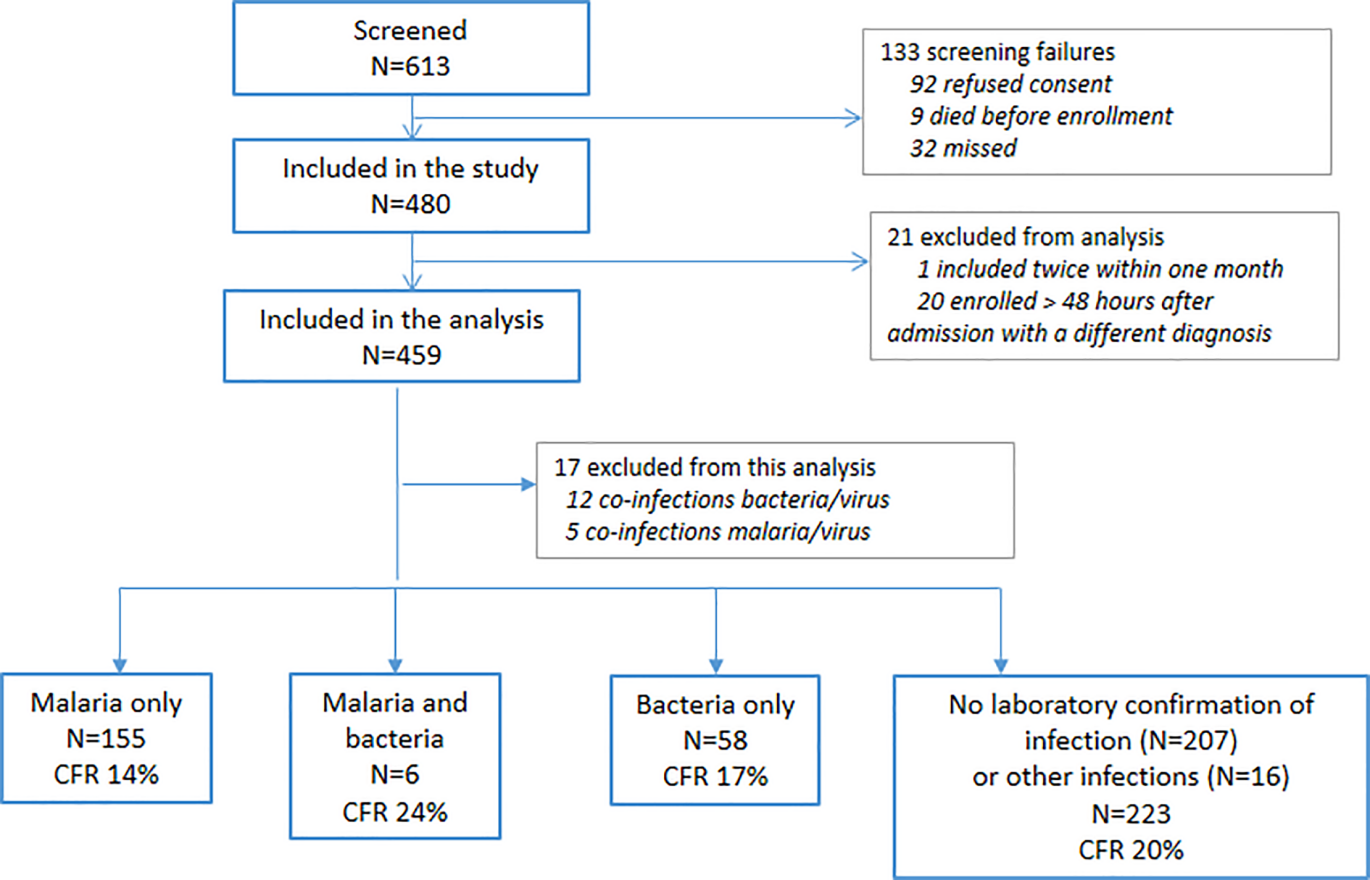
Study flow-chart.

We describe results for 459 children among 480 children with suspected acute CNS infections enrolled in the study (Fig 1); 20 children were excluded because they were enrolled 48 hours after admission, for a diagnosis at admission different from a suspected CNS infection and one child was excluded because he had been included twice in one month.

Of the 459 children with suspected acute CNS infections, 291 (63.4%) were boys. The median age was 30 months (interquartile range (IQR): 11–60). A significant proportion of children presented with reduced consciousness (71.7%) and/or seizures on admission (51.9%). Clinical characteristics at admission are presented in Table 1. Fifty-eight (12.6%) of the children had been hospitalized elsewhere in the month before their admission, for a median duration of 2 days (IQR: 1–4).

**Table 1:** Socio-demographic and clinical characteristics at inclusion of patients overall, survivors and non-survivors by delay between admission and death.

		Total (N = 459)	Survivors (N = 376)	Non-survivors		*p-value
				Died <48h (N = 32)	Died ≥48h (N = 51)	
Patients characteristics						
	Male sex	291 (63.4)	233 (62.0)	23 (71.9)	35 (68.6)	0.38
	Age (months)					0.63
	2–11	122 (26.6)	95 (25.3)	10 (31.3)	17 (33.3)	
	12–59	221 (48.2)	187 (49.7)	13 (40.6)	21 (41.2)	
	≥ 60	116 (25.3)	94 (25.0)	9 (28.1)	13 (25.5)	
	HIV-positive (N = 451)	44 (9.8)	28 (7.6)	3 (9.7)	13 (26.0)	**0.001**
Clinical characteristics at inclusion						
	Reduced consciousness	329 (71.7)	255 (67.8)	31 (96.9)	43 (84.3)	**<0.001**
	Seizures (N = 458)	238 (51.9)	192 (51.1)	21 (65.6)	25 (50.0)	0.284
	Neck stiffness (N = 452)	100 (21.8)	75 (20.0)	4 (13.3)	21 (42.0)	**0.002**
	Hepatomegaly	181 (39.4)	142 (37.8)	18 (56.3)	21 (41.2)	0.117
	Splenomegaly	130 (28.3)	108 (28.7)	13 (40.6)	9 (17.7)	0.071
	Respiratory distress	136 (29.6)	94 (25.0)	18 (56.3)	24 (47.1)	**<0.001**
	Dehydration	80 (17.4)	58 (15.4)	6 (18.8)	16 (31.4)	**0.019**
	Abnormal lung auscultation	63 (13.7)	43 (11.4)	8 (25.0)	12 (23.5)	**0.01**
	Delayed capillary refill	17 (3.7)	10 (2.7)	5 (15.6)	2 (3.9)	**0.006**
	Cold extremities	20 (4.3)	12 (3.2)	4 (12.5)	4 (7.8)	**0.019**
	Cyanosis	4 (0.9)	2 (0.5)	1 (3.1)	1 (2.0)	0.151

* p-value for the comparison between survivors, those who died before 48h and those who died at or after 48h.

The laboratory results confirmed a diagnosis of bacterial and/or malarial infection in 219 (47.7%) children. Malaria was the only confirmed pathogen for 155 (33.8%) children, of which 109 had a clinical diagnosis of cerebral malaria, while 58 (12.6%) children were diagnosed with bacterial infections only, 47 of whom had meningitis; and 6 (1.3%) children had mixed bacterial and malarial infections. Another 17 children had a virus detected in the CSF as a potential co-infection with malaria (n = 5) or bacteria (n = 12) and are not included in the analysis of treatment adequacy due to uncertainty on the etiological agent and lack of viral treatment. Finally, 16 (3.5%) children had another type of infection (tuberculous meningitis, C*ryptococcus*, and/or viruses) and 207 (45.1%) had no laboratory-confirmed diagnosis (more details in [11]).

Overall, 83 children (18.1%) died at the hospital. Of these, over one third (n = 32, 38.6%) died on the day of admission or the next day. There was no significant difference in the proportion who died among those hospitalized in previous month (7/58, 12.1%) and those who were not (76/401, 19.0%). Deaths within 48h of hospitalization were associated with symptoms of severe illness including loss of consciousness, respiratory distress, abnormal lung auscultation or signs of shock (cold extremities and reduced capillary refill), while later deaths were associated with neck stiffness, dehydration and HIV/AIDS (Table 1). Case fatality was 22 (14.2%) for malaria cases, 14 (24.1%) for bacterial cases and 1 (16.7%) with both malaria and bacterial infection.

### Bacteria and antibiotic resistance

Among the 64 children with bacterial infections (including mixed bacterial and malaria infections), the pathogens most frequently isolated were *Streptococcus pneumoniae*, followed by non-typhi *Salmonella* (NTS) and *Haemophilus influenzae* type b (Table 2). Eight children had bacterial meningitis diagnosed based on Gram stain of the CSF; of these, 7 yielded Gram-positive diplococci suggestive of *S*. *pneumoniae*.

**Table 2:** Bacteria isolates from CSF or blood by culture and/or PCR.

	Total	CSF culture & PCR	CSF PCR	CSF culture	Blood culture only
*S*. *pneumoniae*	34	12	15	2	5
*Salmonella* spp.	10	3	3	0	4
*S*. Typhi	3	1	0	0	2
*H*. *influenzae*	5	3	1	1	0
*E*. *coli*	2	0	0	0	2
*S*. *epidemidis*	1	0	0	0	1
*Brevibacterium* spp.	1	0	0	1	0

This table does not include 8 cases for which bacteria were observed in CSF by Gram staining

**Fig 2:**
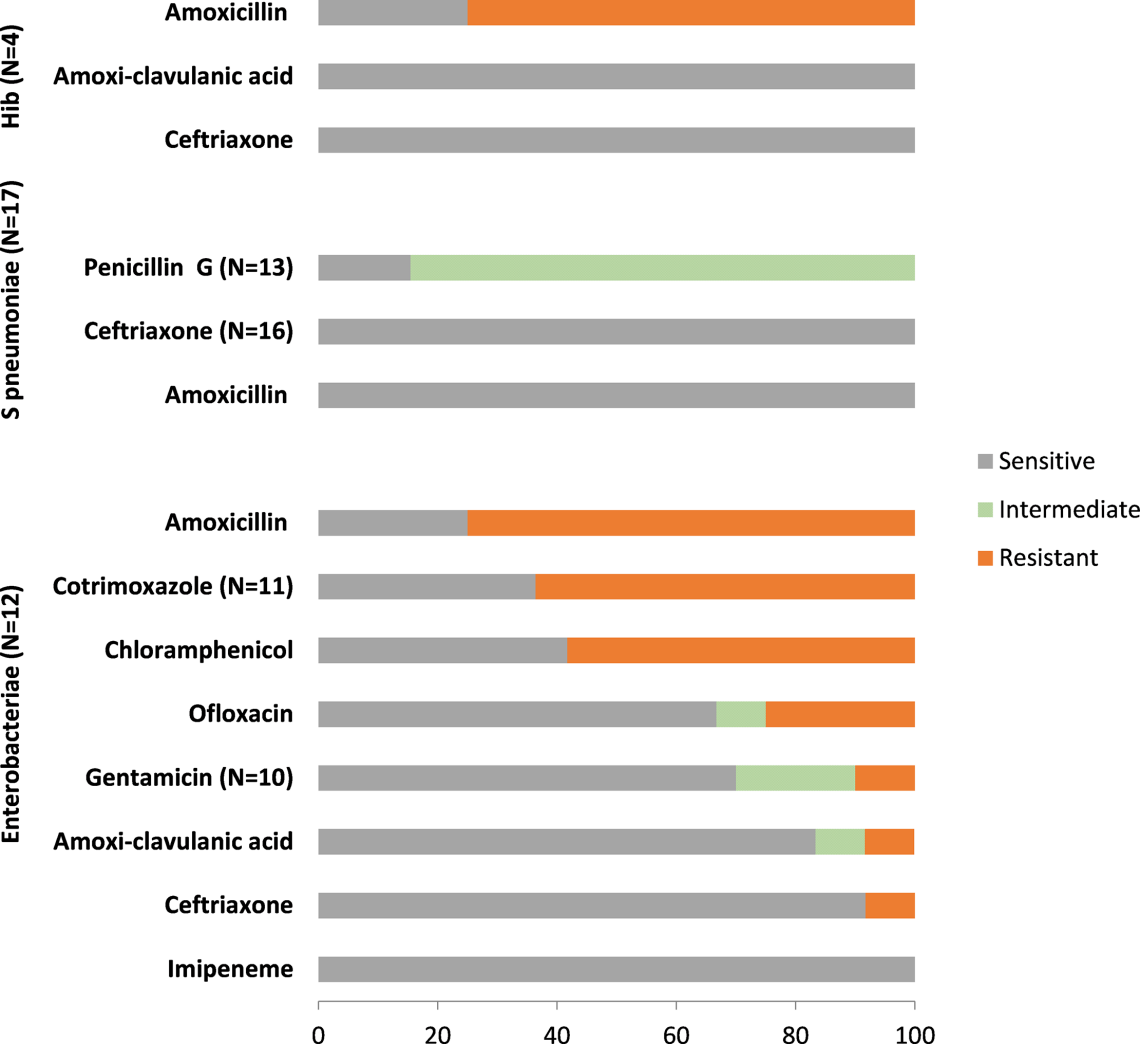
Proportion of sensitive, intermediate and resistant isolates among enterobacteria, *S*. *pneumoniae* and *Haemophilus influenzae* type b isolated in the study.

Twelve enterobacteria (7 NTS, 3 *S*. *typhi*, 2 *E*. *coli*) were tested for antibiotic resistance (Fig 1). Except for one *E*. *coli* with extended beta-lactamase activity (ESBL), the enterobacteria were sensitive to ceftriaxone, but most were resistant to amoxicillin and cotrimoxazole and one third to gentamicin (Fig 2). Notably, all three *S*. *typhi* were resistant to ofloxacin.

All isolates of *S*. *pneumoniae* tested for antibiotic resistance were sensitive to ceftriaxone and amoxicillin, but 11/13 showed intermediate resistance to benzyl penicillin with a mean MIC of 0.34 mg/L. The 4 Hib tested for antibiotic resistance were sensitive to amoxicillin-clavulanic acid and ceftriaxone but 3 were resistant to amoxicillin.

Of the 58 patients who were hospitalized in the previous month, 14 had a bacterial infection (6 *Salmonella* spp, 5 *S*. *pneumoniae*, 2 Hib, 1 *E*. *coli*) but none of these isolates were multidrug resistant.

### Treatments received

Overall, 48.1% (n = 221) of children with suspected CNS infection received antibiotics and/or antimalarial treatment within seven days prior to admission at MRRH or HICH, of whom 42 received antibiotics only, 114 received antimalarials only, and 65 received both. The proportion of patients having received any kind of medication in the seven days prior to admission was higher among children hospitalized in the previous month (77.6% vs 52.1%, p<0.001) and this applied both to antibiotics (39.3% vs 21.5%) and to antimalarials (57.1% vs 37.0%). The main antibiotics received, alone or in combination, prior to admission were benzyl penicillin (n = 44), ceftriaxone (n = 28) and cotrimoxazole (n = 18). The main antimalarials received were quinine (intravenous n = 67, oral n = 36, or intramuscular n = 29) and artemether/lumefrantrine (n = 42).

All but one child, who was rapidly referred to another hospital, received antimicrobial treatment during admission. On the day of admission, 138 (30.1%) children received both antibiotics and antimalarial, 153 (33.3%) children received antibiotics only and 106 (23.1%) antimalarial only. Within 48 hours of admission, an additional 92 children received antibiotics and 39 children received antimalarials.

Among the 291 children who received empiric antibiotic treatment on the day of admission, the most common antibiotics received were ceftriaxone (n = 226, 77.7%) and benzyl penicillin (n = 212, 72.9%), usually as a combination (n = 169, 58.1%). Among those given antimalarial treatment on admission, most of the children (185/244, 75.8%) were treated with quinine, followed by artesunate (n = 51).

### Treatment adequacy

Of the 219 children with laboratory-confirmed bacterial and/or malaria infection, 182 (83.1%) received adequate treatment on the day of admission, while 211 (96.3%) received adequate treatment within 48 hours of admission (Table 1). In total, 8 children did not receive adequate treatment within 48 hours of admission, of which two died. One child with ESBL-producing *E*. *coli* resistant to ciprofloxacin received benzyl penicillin, ceftriaxone and ciprofloxacin within 24 hours of admission but died 48 hours after admission. The other child had a mixed malarial and bacterial infection, was treated with only antimalarials and died within 24 hours of admission before blood culture result was available. There was no significant difference in the proportion of those who received adequate antimalarial or antibiotic treatment at different time points (prior to admission, on the day of admission, or within 2 days of admission) among survivors and non-survivors (Table 3).

**Table 3:** Description of treatment received per type of infection and outcome.

	Survivors	Non survivors	p-value
**Malaria only**	**N = 133**	**N = 22**	
Antimalarials prior admission (N = 153)	66 (51.6)	10 (45.5)	0.57
Antimalarials on admission	111 (83.5)	21 (95.5)	0.20
Antimalarials within 48h of admission	128 (96.3)	22 (100)	1
**Bacterial infection only**	**N = 44**	**N = 14**	
Adequate treatment prior admission (N = 53)	12 (30.0)	2 (15.4)	0.47
Adequate treatment on admission	36 (81.8)	12 (85.7)	1
Adequate treatment within 48h of admission	43 (97.7)	13 (92.9)	0.43
**Mixed malaria and bacterial infection**	**N = 5**	**N = 1**	
Adequate treatment prior admission	0 (0)	0 (0)	/
Adequate treatment on admission	2 (40.0)	0 (0)	1
Adequate treatment within 48h of admission	5 (100)	0 (0)	0.17

### Treatment duration

Of the 161 children who had malaria (including 6 with mixed malaria and bacterial infection), 9 (5.6%) children had an insufficient duration of treatment and 17 (10.6%) an excessive duration. Among 58 children with bacterial infections only, 7 (12.1%) had an insufficient duration of treatment (5 to 8 days), while 6 (10.3%) had an excessive duration. Finally, 2 of 6 with mixed malaria and bacterial infection had an insufficient duration of treatment.

## Discussion

In the context of global rise in antibiotic resistance [16,17] and the absence of microbiology capacity in most resource-limited settings (11), clinicians face uncertainty regarding the appropriateness and efficacy of empirical treatments. This study of the aetiology of CNS infections in Mbarara provided us with an opportunity to investigate the adequacy of treatment received by children admitted with bacterial or malaria infection. We found a high mortality in children, particularly among those with bacterial CNS infection, in line with other findings from Africa reporting in-hospital CRFs ranging from 9% to 67% for all types of confirmed bacterial meningitis [3] and from 18% to 94% for pneumococcal meningitis [18]. In the present study, all meningitis deaths but two had received adequate treatment within 48h of admission, suggesting that lack of adequate treatment in the hospital was not the main cause of the high mortality, while illustrating some of the main challenges for empiric treatment in these contexts: antibiotic resistance and co-infections.

The levels of antibiotic resistance among isolates found in this study was relatively low, in line with Ugandan data reported in the 2014 Antimicrobial Resistance report published by WHO [17]. Though reduced susceptibility of *S*. *pneumoniae* to benzyl penicillin was already reported in children with pneumococcal meningitis in Uganda in 2005–2006 [19], no complete resistance to penicillin has been observed here or elsewhere in this area [20]. Given the antibiotic resistance patterns with reduced susceptibility to benzyl penicillin and presence of enterobacteria as causative agents in some children, it is reasonable to use ceftriaxone or cefotaxime as first line management for acute meningitis in this region empirically, as recommended in the Ugandan clinical guidelines [15]. However, one of the two children who died without receiving adequate treatment was infected with an ESBL-producing multidrug resistant *E*.*coli* isolate. This raises a concern as such infection is difficult to detect and active agents against ESBL-producing gram-negatives, such as carbapenems, were not available on site for treatment and remain largely unavailable in the African public sector. Hence, close monitoring of the possible emergence of ESBL enterobacteria is warranted.

The other child who died without receiving adequate treatment had a mixed bacterial-malaria infection but was treated for malaria only. The current WHO recommendation is to administer broad-spectrum antibiotics to children with severe malaria until a bacterial infection is excluded [21]. However, these recommendations are debated considering the high prevalence of malaria and consequences of adding an antibiotic both in terms of cost and risk for antibiotic resistance [22]. It also shows the need to develop rapid tests for identifying life-threatening bacterial infections occurring with or without malaria.

Although the majority of children, including those who died, had received adequate treatment within 48h of admission, the time of administration of antimicrobial treatment was not optimal for all children. The delay in initiation of treatment could be due to delay in clinical decision making by junior medical officers or drug shortages, as commonly seen in this context. Refresher training and supervision of junior health workers and nursing staff to adhere to treatment protocols could be helpful in ensuring prompt administration of antimicrobial treatment. Indeed, almost one in five children did not receive adequate treatment on the day of hospital admission. The proportions were similar in children who survived and died, suggesting no direct association with increased risk of death, however there may be other factors involved that could have obscured such a relationship.

We found both insufficient duration of antibiotics in some children and excessive duration in others. Insufficient duration may place children at higher risk of death and of relapse, especially in low-resources settings where follow-up outpatient care systems are weak. Under-treatment is also a concern with pathogens that are more difficult to eradicate including *Salmonella* spp. [23]. Here, however, all children with insufficient duration of antibacterial treatment had received at least 5 days of treatment, which was shown to be sufficient in most cases [24]. Over-treatment comes with other risks including the risk to patients of prolonged hospitalization, elevated risk of treatment-related toxicity, inconvenience / loss of income for family and the potential contribution to individual and community antibiotic resistance. Non-adherence to treatment guidelines in humanitarian hospitals has been associated with poor outcomes such as death or loss to follow up in pediatric patients [25].

One important limitation of this study was that we did not have data on duration of symptoms prior to seeking medical care or hospitalization; delayed presentations could have resulted in increased mortality. We also did not have data on the precise time of treatment initiation upon presentation, although the majority of patients received treatment on the day of admission. Another limitation was the inclusion of children with various clinical presentations at admission and types of infections, including cerebral and non-cerebral malaria, bacterial meningitis and bacteremia with neurological symptoms, with different risks of death. However, this reflects the practical reality of clinical medicine in a pediatric hospital in Uganda. Furthermore, the categorization of treatment adequacy is challenging and we did neither consider treatment dosages nor the additional (non-antimicrobial) treatments received by the children. Finally, this study was a secondary analysis of a pre-existing cohort and may not have been adequately powered to detect differences in the outcomes of interest. Therefore, the findings should be considered to be exploratory.

Despite these limitations, the data suggest that it is unlikely that the high in-hospital mortality observed in this study was due to inadequate initial antimicrobial treatment. Hence, we speculate that some of the contributing factors could have been delay in presentation from poor treatment access or late caregiver disease recognition, severity of disease or late administration of the antimicrobial on the day of admission [26]. This highlights the need for better recognition and management of severe febrile illnesses in the community. Given the high prevalence of bacterial meningitis among children admitted with fever and suspicion of CNS infection and local patterns of antibiotic susceptibility, the empirical use of a third-generation cephalosporin on admission appears to be justified in this setting, but the evidence of emerging resistance to cephalosporins among enterobacteria emphasizes the need for close monitoring of antibiotic resistance.

## Supporting information

S1 Dataset Study dataset. All dates in the dataset were randomly modified to ensure data anonymization.
(XLSX)
